# Primary peritoneal drainage in neonates with necrotizing enterocolitis associated with congenital heart disease: a single experience in a Brazilian tertiary center

**DOI:** 10.1590/1414-431X2020e10220

**Published:** 2021-05-31

**Authors:** W.C. Canesin, F.A.P. Volpe, W.A. Gonçalves-Ferri, P.H. Manso, D.C. Aragon, L. Sbragia

**Affiliations:** 1 Laboratório de Cirurgia Experimental Fetal “Michael Harrison”, Divisão de Cirurgia Pediátrica, Departamento de Cirurgia e Anantomia, Faculdade de Medicina de Ribeirão Preto, Universidade de São Paulo, Ribeirão Preto, SP, Brasil; 2 Departamento de Pediatria, Faculdade de Medicina de Ribeirão Preto, Universidade de São Paulo, Ribeirão Preto, SP, Brasil

**Keywords:** Necrotizing enterocolitis, Peritoneal drainage, Congenital heart diseases

## Abstract

Necrotizing enterocolitis (NEC) is a common condition in preterm infants. The risk factors that contribute to NEC include asphyxia, apnea, hypotension, sepsis, and congenital heart diseases (CHD). The objective of this study was to evaluate the association between the treatment (surgery or drainage) and unfavorable outcomes in neonates with NEC and congenital heart diseases (NEC+CHD). A 19-year retrospective cohort study was conducted (2000-2019). Inclusion criterion was NEC Bell II stage. Exclusion criteria were associated malformation or genetic syndrome and those who did not undergo echocardiography or had a Bell I diagnosis. We included 100 neonates: NEC (n=52) and NEC+CHD (n=48). The groups were subdivided into NEC patients undergoing surgery (NECS, n=31), NEC patients undergoing peritoneal drainage (NECD, n=19), NEC+CHD patients undergoing surgery (NECCAS, n=21), and NEC+CHD patients who were drained (NECCAD, n=29). Multivariate analysis was performed to estimate the relative risk of death and the length of stay. Covariates were birth weight and gestational age. The group characteristics were similar. The adjusted relative risk of death was higher in the drainage groups [NECD (Adj RR=2.70 (95%CI: 1.47; 4.97) and NECCAD (Adj RR=1.97 (95%CI: 1.08; 3.61)], and they had the shortest time to death: NECD=8.72 (95%CI: 3.10; 24.54) and NECCAD=5.32 (95%CI: 1.95; 14.44). We concluded that performing primary peritoneal drainage in neonates with or without CHD did not improve the number of days of life, did not decrease the risk of death, and was associated with a higher mortality in newborns with NEC and clinical instability.

## Introduction

Necrotizing enterocolitis (NEC) is the most common reason for neonatal abdominal surgery. The overall incidence of NEC is approximately 1 in 1000 live births, but its incidence is clearly inversely related to the birth weight and gestational age, and it affects up to 10% of infants weighing less than 1500 g (1). NEC has a multifactorial etiology, and an inadequate inflammatory response to some type of insult (congenital heart disease, infection, formula feeding, or combined factors) may culminate in intestinal necrosis and perforation (2).

Full-term infants who develop NEC usually have other associated factors that predispose them to the disease, such as sepsis, low Apgar scores, prolonged rupture of membranes, exchange transfusions, neural tube defects, and congenital heart disease (CHD) (3). Patients with CHD have a 3.3-11% risk of developing NEC (4,5), which is much higher relative to other full-term infants. The pathophysiology of NEC in CHD patients remains unknown, but it is theorized that infants with CHD have lower diastolic pressure, which leads to lower bowel perfusion pressures and lower systemic oxygenated blood flow, contributing to an overall state of bowel hypo-perfusion and increased levels of circulating pro-inflammatory cytokines (6).

NEC is initially treated through clinical management, but surgical intervention is required if intestinal perforation or necrosis occurs (7). Mortality by surgical NEC is still approximately 50%, despite all of the scientific improvements in the last few decades (8). However, some authors advocate that surgical intervention results in higher survival than medical management in patients with CHD (9). Surgical treatment may be achieved by laparotomy or primary peritoneal drainage (PPD) (10), but which is the best technique is still unclear (11,12). In fact, adequate evidence regarding the best treatment for NEC in babies with hemodynamic instability and patients with patent ductus arteriosus (PDA) or other heart diseases is lacking.

We aimed to evaluate the association between the type of treatment (drainage or surgery) and outcomes in patients with NEC and CHD.

## Material and Methods

This study was approved by the local ethics committee for human research approval number - CEP/Plataforma Brasil CAAE: 16180819.8.0000.5440 (number: 077334/2019, Ribeirao Preto Medical School, USP). We retrospectively analyzed the medical charts of newborns hospitalized at a Ribeirão Preto general hospital from 2000 to 2019. The inclusion criterion was a diagnosis of NEC (Bell II or III) (13) made by the neonatology staff. The exclusion criteria were other malformations, a genetic syndrome, those who did not undergo echocardiography, or had a Bell I diagnosis. All of the NEC and NEC+CHD patients were submitted to an echocardiogram performed by the cardiology staff. We considered heart disease to be a PDA >1 cm and other congenital heart defects.

The patients were divided into two groups depending on the presence of CHD (the NEC and NEC+CHD groups). The groups were subdivided into NEC undergoing surgery (NECS), NEC undergoing peritoneal drainage (NECD), NEC+CHD undergoing surgery (NECCAS), and NEC+CHD undergoing drainage (NECCAD).

In the groups with drainage, the PPD was performed in the lower right quadrant when the babies had pneumoperitoneum and could not be immediately submitted to surgery due to severe thrombocytopenia, hemodynamic instability, bleeding, or shock with a demand for vasoactive drugs. As soon as the baby achieved clinical stability, they would be submitted to laparotomy.

In the groups with surgery, a laparotomy was performed if the baby had pneumoperitoneum or showed no improvement after 15 days of clinical treatment (fasting, abdominal decompression, and broad-spectrum antibiotics).

Chorioamnionitis and maternal hypertension diagnoses were performed by the obstetricians. Small to gestational age was determined by Intergrowth 21. Gestational age was estimated by the Ballard score for newborns <37 weeks and the Capurro score for ≥37 weeks.

For the estimation of gross and adjusted relative risks, simple and multiple log-binomial regression models were used. Kaplan-Meier curves were constructed to describe the length of stay. The time until death and the time until discharge from the ICU were evaluated. For the latter, deaths were removed from the database. For the time until death, hazard ratios were estimated by adjusting a Cox proportional hazards model. In this case, weight, gestational age, and heart disease (only for comparisons of 2 groups) were also considered as covariates. For the time until discharge from the ICU, the groups were compared using the Wilcoxon test.

## Results

We included 273 patients with NECII in the period studied, and after applying the exclusion criteria, 173 patients were excluded. We analyzed 100 patients, 48 (48%) of which had NEC and CHD.

Table 1 shows the characteristics of the groups. We noticed similar characteristics among the groups, although chorioamnionitis was more predominant in the groups with CHD. The length of stay was the same among the groups.

**Table 1:** Characteristics of the studied groups according to the presence of heart disease and type of surgical treatment.

Characteristics	NECS N=31	NECCAS N=19	NECD N=21	NECCAD N=29	P
Birth weight in g (mean/SD)	1227.2 (359)	1252.89 (661)	1054.52 (274)	1213.90 (664)	0.37
Gestational age in weeks (mean/SD)	31.4 (2.9)	30.5 (3.9)	30.0 (2.8)	30.0 (4.1)	0.10
Male (n, %)	16 (51.6)	12 (63.1)	9 (42.8)	16 (55.1)	0.63
Maternal hypertension (n, %)	11 (35.4)	4 (21.0)	3 (14.2)	3 (10.3)	0.10
Antenatal steroids (n, %)	15 (48.3)	8 (42.1)	11 (52.3)	10 (34.4)	0.67
Small for gestational age (n, %)	18 (58.0)	5 (26.3)	10 (47.6)	9 (31.0)	0.68
Chorioamnionitis (n, %)	6 (19.3)	11 (57.8)	6 (28.5)	12 (41.3)	0.04
Length of stay (mean/SD)	78.5 (52.8)	120.2 (104.9)	40.9 (31.1)	70.7 (82.5)	0.63

CHD: congenital heart disease; NECS: necrotizing enterocolitis (NEC) patients undergoing surgery; NECD: NEC patients undergoing peritoneal drainage; NECCAS: NEC+CHD patients undergoing surgery; NECCAD: NEC+CHD patients who were drained. ANOVA or chi-squared test.

A multivariate analysis was performed to evaluate the association between death and the presence of heart disease and the type of surgical treatment. There was an association between death and drainage in both groups; however, the adjusted relative risk was higher in the group without heart disease (Table 2).

**Table 2:** Number (%) of deaths in the groups and relative risk (RR) according to the presence of heart disease and type of surgical treatment.

Characteristics	Death	Survival	RR
NECS N=31 (%)	6 (19.3)	25 (80.6)	1.45 (0.66; 3.21)
NECCAS N=19 (%)	6 (31.5)	13 (68.4)	2.70 (1.47; 4.97)
NECD N=21 (%)	15 (71.4)	6 (28.5)	1.97 (1.08; 3.61)
NECCAD N=29 (%)	21 (72.4)	8 (27.5)	1.06 (0.73; 1.54)

Covariates: birth weight and gestational age. CHD: congenital heart disease; NECS: necrotizing enterocolitis (NEC) patients undergoing surgery; NECD: NEC patients undergoing peritoneal drainage; NECCAS: NEC+CHD patients undergoing surgery; NECCAD: NEC+CHD patients who were drained. Simple and multiple log-binomial regression analysis.

**Figure 1:**
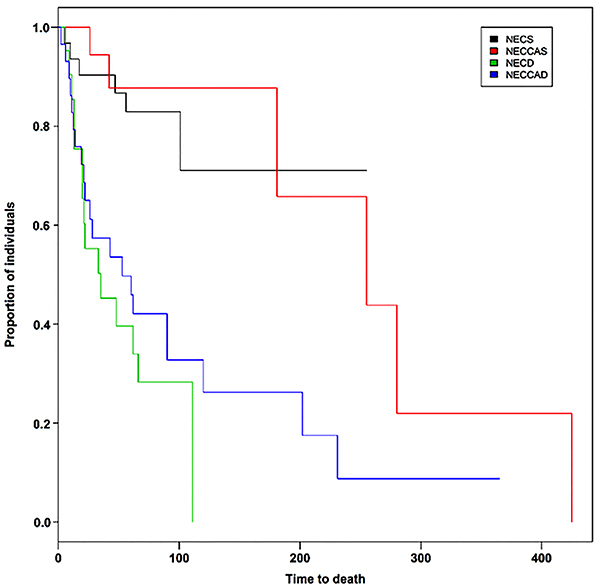
Time to death (days) in the groups according to the presence of heart disease and type of surgical treatment. CHD: congenital heart disease; NECS: necrotizing enterocolitis (NEC) patients undergoing surgery; NECD: NEC patients undergoing peritoneal drainage; NECCAS: NEC+CHD patients undergoing surgery; NECCAD: NEC+CHD patients who were drained.

We assessed the time to death in the newborns according to the presence of heart disease, type of surgical treatment, and gestational age (Figure 1) and observed that the shortest time to death occurred in the NECD group.

Table 3 shows the association between time to death and the groups. The patients submitted to drainage had the highest risk of death and their deterioration was faster compared with NECS.

**Table 3:** Association between time to death and the groups in the non-survivor patients.

Groups	Hazard ratio (95%CI)	Hazard ratio (95%CI) - adjusted
NECS	Reference	Reference
NECCAS	0.94 (0.28; 3.13)	1.14 (0.32; 4.09)
NECD	6.10 (2.34; 15.84)	8.72 (3.10; 24.54)
NECCAD	4.21 (1.69; 10.53)	5.32 (1.95; 14.44)

Covariates: birth weight and gestational age. CHD: congenital heart disease; NECS: necrotizing enterocolitis (NEC) patients undergoing surgery; NECD: NEC patients undergoing peritoneal drainage; NECCAS: NEC+CHD patients undergoing surgery; NECCAD: NEC+CHD patients who were drained.

## Discussion

Our data demonstrated the worst outcomes in newborns submitted to drainage in both groups. Drainage did not avoid a higher incidence of death in patients with surgical NEC and clinical instability in the newborns with NEC or NEC+HCD.

The group characteristics were similar, and the HCD presence and the type of treatment performed did not affect the length of stay. There is a consensus that occurrence of NEC has worse outcomes in neonates with CHD. Regarding heart diseases, the persistence of the ductus arteriosus, use of indomethacin to close the ductus, umbilical catheterization, and CHD are possible triggers for NEC development, which affects between 7 and 20% of this population (3,14–19).

In a case-control study, Bubberman et al. (20) compared 18 NEC+CHD and 36 NEC patients and found different postnatal ages at the onset of symptoms, with an earlier onset in NEC+CHD. The pH levels were lower and the C-reactive protein levels were higher in NEC. In addition, the anatomical location was different: the colon was more frequently affected in NEC+CHD (86 *vs* 33% in NEC, P=0.03). However, these authors did not find any difference in mortality between the groups (22 *vs* 11%, respectively, P=0.47).

NEC pathophysiology in cardiac patients results from low blood flow in the mesenteric vessels, a consequence of reverse flow in the abdominal aorta secondary to low diastolic pressure through the hypoxia compensatory mechanism called the “diving reflex” (15). Cardiac patients with ascites often require paracentesis, with hemodynamic improvement (16 [Bibr B17] [Bibr B18]). Some types of heart disease, such as left ventricular hypoplasia, a single ventricle, coarctation of the aorta, and arteriovenous truncus, represent an even higher risk for NEC (3). The best approach to the treatment of NEC+CHD needs to be clarified, especially in neonates with clinical instability. Is PPD associated with better outcomes in these patients?

The conventional treatment of neonates with perforation or intestinal necrosis is laparotomy, with the creation of an ostomy. In newborns, this treatment poses a tremendous surgical risk due to the patient’s critical condition (21). PPD without posterior laparotomy is related to a higher mortality and longer hospitalization than laparotomy alone (8), so its use as a definitive treatment method is not justified. Unfavorable PPD results include the presence of an intestine with necrosis and a predisposition to sepsis, which stimulates cytokine release and negatively affects the organism, deteriorating the clinical condition of the newborn (22).

Ehrlich et al. (23) analyzed the surgical procedures performed on 70 newborns weighing less than 1,000 g who developed NEC, and they observed a survival rate of 63 and 75% in babies that underwent PPD and laparotomy, respectively. However, patients submitted to PPD had a significantly higher number of complications. Through regression analysis, the authors concluded that the surgical choice did not influence the results. Recently, Moss et al. (24) performed a meta-analysis of studies that used both procedures to treat children with NEC. The analysis included 475 patients and showed no significant difference between laparotomy and PPD for mortality. Other studies also did not verify any significant difference in morbidity, mortality, or the length of hospital stay in newborns undergoing PPD followed by laparotomy compared with those undergoing isolated laparotomy (25). In addition, most newborns subjected to PPD required laparotomy as a second step (11,26).

However, Tashiro et al. (27) reported that PPD proved useful as an initial surgical treatment in hemodynamically unstable premature patients weighing less than 1000 g. They demonstrated patient survival was higher in NEC+CHD that underwent PPD (47%) than in NEC patients in general (10%). Among patients undergoing PPD, patients with heart disease had higher survival rates. The lack of improvement in the survival of newborns diagnosed with NEC was influenced by nonspecific and often random surgical and therapeutic strategies (16).

In our study, PPD was associated with death in NECS and NECD as well as in NECCAS and NECCAD. The relative risk of death in NECCAD was higher than in NECCAS, and it was also higher in NECD compared with NECS; however, the neonates without heart disease presented a higher risk of death than newborns with heart disease. The lower risk of these patients can be explained by their more significant hypoxemia, which could partially represent ischemic preconditioning and, therefore, serve as a protective state against intestinal damage (28).

Our data found an association with faster progression to death in neonates submitted to drainage, but these data have limitations since our indication for drainage was clinical instability, and the worse outcomes are probably related to their clinical conditions. Therefore, our data are in accordance with the literature and demonstrated that drainage was ineffective in both groups and was associated with worse outcomes.

### Conclusion

In conclusion, primary peritoneal drainage in neonates with or without CHD did not improve days of life, did not decrease the risk of death, and was associated with higher mortality in newborns with NEC and clinical instability.
